# Determination of biogenic amines by high-performance liquid chromatography (HPLC-DAD) in probiotic cow's and goat's fermented milks and acceptance

**DOI:** 10.1002/fsn3.200

**Published:** 2015-01-22

**Authors:** Marion P Costa, Celso F Balthazar, Bruna L Rodrigues, Cesar A Lazaro, Adriana C O Silva, Adriano G Cruz, Carlos A Conte Junior

**Affiliations:** 1 Department of Food Technology, Universidade Federal FluminenseRio de Janeiro, Brazil; 2 Department of Animal Health and Public Health, Universidad Nacional Mayor de San MarcosLima, Peru; 3 Master in Food and Science Program, Instituto Federal de Educação, Ciência e Tecnologia do Rio de JaneiroRio de Janeiro, Brazil

**Keywords:** Cadaverine, cow's milk, fermented milks, goat's milk, lactic acid bacteria, putrescine, tyramine

## Abstract

This study evaluated the presence of biogenic amines in fermented cow's and goat's milks containing probiotic bacteria, during the first 10 days of chilled storage (4 ± 2°C), when the probiotic strains are most viable. The overall acceptance of both fermented milks, produced using the same starter culture and probiotics, was tested. In both products, the initially high levels of tyramine (560 mg kg^−1^ means for both fermented milks), the predominant biogenic amine, increased during the storage period, which may be considered this amine as a quality index for fermented milks. The other principal biogenic amines (putrescine, cadaverine, histamine, and spermidine) were produced on days 1–5 of storage, and thereafter decreased. At the end of the 10th day, these amines, respectively, showed values of fermented cow's milk 20.26, 29.09, 17.97, and 82.07 mg kg^−1^; and values of fermented goat's milk 22.92, 29.09, 34.85, and 53.85 mg kg^−1^, in fermented cow's and goat's milk. Fermented cow's milk was well accepted compared to fermented goat's milk. The results suggested that the content of biogenic amines may be a criterion for selecting lactic acid bacteria used to produce fermented milks.

## Introduction

Fermented milks are a traditional food, and the use of goat's and cow's milks as raw materials is well established in the modern dairy industry (Tamime et al. 2011; Costa et al. 2014). The addition of probiotic bacteria to these products adds value with respect to their potential functional benefits. Thus, fermented milks have high potential for the development of new products, mainly due to their association with health and well-being (Costa et al. 2013). Mixed cultures, such as *Lactobacillus acidophilus* and *Bifidobacterium lactis*, have been successfully used in dairy products (Buriti et al. 2007, 2010; Costa and Conte-Junior 2013). During the first 10 days of storage, the probiotic strains are highly viable in both fermented milks (cow and goat) (Varga et al. 2014). At this stage, probiotics produce substances that may provide beneficial effects to human health (Kongo et al. 2006; Conte-Junior et al. 2007). However, other kinds of metabolics, such as biogenic amines, may also be produced by probiotic strains during this period.

Biogenic amines can be formed in food during processing or the period of storage, primarily due to the release of specific amino acids by the action of decarboxylases produced by microorganisms. Certain bacterial genera, with potential probiotic characteristics, can form biogenic amines (Priyadarshani and Rakshit 2011). Ingestion of foods containing high levels of biogenic amines, such as tyramine and histamine, may be deleterious, since these amines have vasoactive, psychoactive, and toxicological properties. In addition, putrescine and cadaverine may potentiate the toxicity of these biogenic amines (Flick et al. 2001). The presence and accumulation of these substances are influenced by numerous factors, such as the composition and availability of free amino acids, water activity, temperature, the pH of the medium, and especially the presence of decarboxylase-positive microorganisms (Schirone et al. 2012).

The production and storage of fermented milks favor the formation of biogenic amines by augmenting the activity of proteolytic microorganisms, which increases the amount of free amino acids (Linares et al. 2011). The types and contents of biogenic amines present in fermented dairy products vary with the feedstock, product type, ripening/fermentation time, culture starter strains, proteolytic activity, and manufacturing conditions (Andic et al. 2010; Priyadarshani and Rakshit 2011).

The purpose of this study was to evaluate the presence of the biogenic amines tyramine, putrescine, cadaverine, spermidine, and histamine, in probiotic fermented cow's and goat's milks during the first 10 storage days at 4 ± 2°C, when the viability of probiotic strains is highest (Kongo et al. 2006). Additionally, a sensory test was carried out in order to assess the products' overall acceptability.

## Materials and Methods

### Fermented milk processing

The fermented milks were prepared using UHT cow's and goat's milks (cow's milk from Macuco®, Rio de Janeiro, Brazil; goat's milk from Caprilat®, Paraná, Brazil). To produce the both fermented milks 4 × 10^8^ CFU mL^−1^ lyophilized *Lactobacillus acidophilus* LA-5®, *Bifidobacterium lactis* BB-12®, and *Streptococcus thermophilus* (Chr Hansen, Valinhos, Brazil) cultures were added in DVS form (direct vat set). Subsequently, the samples remained in an oven for 8 h at 42 ± 1°C, for the fermentation process, until the pH reached 4.6. Then, fermentation was stopped by refrigerating the fermented milks at 5 ± 1°C. Finally, the product was homogenized, fractionated, and packed in 200-mL plastic pots and stored at 4 ± 1°C.

### Physicochemical analyses

Samples of the fermented milks were analyzed for pH (AOAC 2012) and biogenic amines (Cunha et al. 2012) when immediately after fermentation (day 0), and each day during the first 10 days of chilled storage (4 ± 2°C), when the probiotic strains are most viable (Kongo et al. 2006). For pH analyses, a digital pH meter (pH Model PG1800, Cap Lab®, São Paulo, Brazil) was used. This experiment was replicated two times, and all analyses were performed in triplicate.

Biogenic amines were identified and the quantitative by high-performance liquid chromatography (HPLC), which extraction and derivatization were according to methodology described by Cunha et al. (2012). For the extraction, 5 g of fermented milks was homogenized with 5 mL of 5% perchloric acid. The samples were kept under refrigeration (4 ± 2°C) for 1 h and shook continuously every 10 min. After that, the mixture was centrifuged at 1000 *g* for 10 min at 4 ± 1°C (Hermle Z 360 K) and filtered through Whatman no. 1 filter paper (180 *μ*m thickness and 11 *μ*m particle retention rating at 98% efficiency). The pH of filtrates were neutralized with 2 N NaOH and kept in an ice bath (0 ± 2°C) for 20 min, followed by a second filtration, and addition of NaOH (pH > 12) under the same conditions. For the derivatization, 40 *μ*L of benzoyl chloride was added and kept at room temperature for 20 min. The mixture was extracted with diethyl ether, which was aspirated and evaporated to dryness under a stream of nitrogen (Sample Concentrator Techne®, Cambridge, UK). Finally, the residue was dissolved in acetonitrile: water 42:58 (% v/v) and stored at 4 ± 1°C.

A Shimadzu® model LC/10 AS, coupled to UV detector SPD/10 AV, chromatographic system was used with C-R6A Chromatopack integrator, using a Teknokroma column, Extrasil Tracer ODS2 (15 × 0.46 cm, id. 5 mm) and Supelco pre column, C18 Ascentis (2 × 0.40 cm, id. 5 *μ*m). Exactly 20 *μ*L of the prepared sample was injected into the HPLC. The mobile phase consisted of acetonitrile: water 42:58 (% v/v), which was performed isocratically at 1.0 mL min^−1^ flow rate. Peaks were detected at 198 nm.

**Figure 1:**
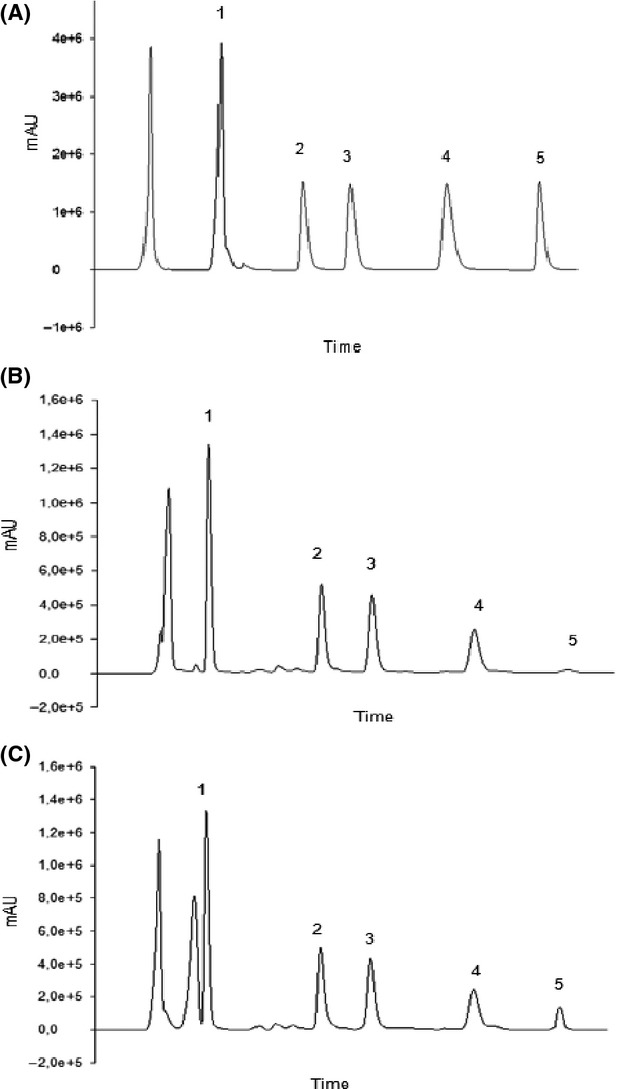
HPLC chromatograms relative to: (A) Standard solution of five biogenic amines; (B) Cow's fermented milk sample; (C) Goat's fermented milk sample. Biogenic amines and retention times, respectively: 1. tyramine (2.85); 2. putrescine (4.67); 3. cadaverine (5.71); 4. spermidine (7.85); 5. histamine (12.59).

Five biogenic amines were quantified: tyramine (C_8_H_11_NO), putrescine (C_4_H_12_N_2_), cadaverine (C_5_H_14_N_2_), spermidine (C_14_H_47_N_6_O_12_P_3_), and histamine (C_5_H_9_N_3_). Biogenic amines standards were purchased from Sigma-Aldrich® (St. Louis, MO). Stock solutions for each amine were prepared in 0.1 N HCl and stored at 4 ± 1°C. For amine identification, standard solutions of individual biogenic amines were chromatographed separately and mixed to determine the retention times and the response of each (Fig.1). Standard curves with correlation coefficients for stock solutions were obtained by the external standard method. All the results were expressed in mg kg^−1^.

### Consumer test

The sensory evaluation of the fermented milks (cow and goat) was performed 1 day after their manufacture, and the overall acceptance was assessed by a hedonic test. A 9-point hedonic scale was used, with 60 consumers ranging from 17 to 61 years old (37 females and 23 males) participating (Drake 2007). These panelists consisted of students, randomly recruited from the Fluminense Federal University, Brazil. The inclusion criterion was regular consumption of dairy products, while people with an allergy or intolerance to dairy products were not recruited.

The sensory analysis was performed on the next day of fermented milks production. The 20 mL samples were coded with three-digit codes and presented monadically according to a randomized complete block design (Macfie et al. 1989). The test was performed by panelists in individual booths. They were asked to evaluate the overall acceptability of the fermented milks, based on a 9-point hedonic scale: like extremely = 9, like very much = 8, like moderately = 7, like slightly = 6, neither like nor dislike = 5, dislike slightly = 4, dislike moderately = 3, dislike very much = 2, dislike extremely = 1.

### Statistical analysis

The results of the physicochemical and sensory tests were subjected to one-way analysis of variance (ANOVA) followed by Tukey's test. The content of tyramine and days of storage was subjected to Pearson correlation analysis. All analyses were performed using XLSTAT software (version 2013.2.03; Addinsoft, Paris, France). A *P*-value below 5% (*P* < 0.05) was regarded as significant.

## Results and Discussion

### Physicochemical analyses

The initial pH values of the cow's and goat's milks (6.71 and 6.70, respectively, *P* < 0.05) were reduced, respectively, to 4.51 and 4.48 after the fermentation process ended. These final pH values are in line with the growth of the starter culture and probiotic bacteria. This evolution might be due to lactose fermentation, which produces lactic acid and lowers the pH. During storage, the mean pH value was 4.50 for fermented cow's milk and 4.51 for fermented goat's milk (*P *> 0.05), suggesting that there was no post acidification. This finding was probably due to the absence of *Lactobacillus delbrueckii bulgaricus* in the fermented milks, because this bacterium is responsible for post acidification (from lactic acid and hydrogen peroxide) during refrigerated storage (Cruz et al. 2013).

The pH is an important factor for fermentation and the formation of biogenic amines, because amino acid decarboxylase activity is higher in an acidic environment. This may explain why decarboxylase enzymes have an optimum pH of around 5.0. Furthermore, the bacterial growth also increases the amount of biogenic amines, by raising the production of the decarboxylase enzyme (Lázaro et al. 2013).

In the determination of biogenic amines using high-performance liquid chromatography, standard curves with correlation coefficients of 0.9981 (tyramine), 0.9977 (putrescine), 0.9997 (cadaverine) 0.9921 (spermidine), and 0.9343 (histamine) were obtained by the external standard method. All five biogenic amines were well separated with good peak resolution, sharpness, and symmetry (Fig.1A). Regarding the limit of detection (LOD) and limits of quantification (LOQ), the amines studied ranged from 0.03 to 1.30 mg L^−1^ and 0.20 to 5.00 mg L^−1^, respectively. In addition, the recovery for these amines ranged from 91% to 107%. The Table1 presents biogenic amine contents (mg kg^−1^) and total concentration in both fermented milks (goat and cow), on the 10th day.

**Table 1:** Biogenic amine contents (mg kg^−1^), values of fermented milks samples, on the 10th day

Biogenic amines	Goat's fermented milk	Cow's fermented milk
Tyramine	337.11	249.55
Putrescine	22.92	20.26
Cadaverine	22.07	29.09
Histamine	53.85	17.97
Spermidine	34.85	82.07
Total concentration	470.80	398.94

During the first 10 days of storage at 4 ± 2°C, tyramine was the most abundant amine present in both fermented milks. This result is in accordance with the contents found in other dairy products (Andic et al. 2010; Özdestan and Üren 2010). Tyramine is the most commonly detected biogenic amine in fermented dairy products, since many lactic acid bacteria can produce microbial tyrosine decarboxylase (Buňková et al. 2010), which explains the high values of tyramine found in this study.

**Figure 2:**
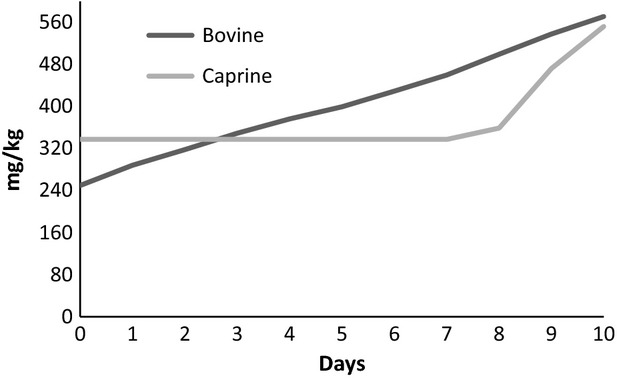
Tyramine behavior found in both fermented milk (cow and goat) during 10 days of storage.

Up to the third storage day, the tyramine content was higher in the fermented goat's milk than in the fermented cow's milk, reaching the levels of 337.11 and 249.55 mg kg^−1^, respectively. In goat's milk, the content of this amine remained stable until the seventh day, thereafter presented an increase. In cow's milk, tyramine increased linearly during storage (Fig.2) presenting a positive correlation between tyramine increase and days of storage (*r* = 0.99; *P *< 0.05). Thus, tyramine may be considered as a quality index for fermented milks. The different behavior of fermented cow's and goat's milk up to the eighth day might be correlated mainly with: different protein compositions, particularly the casein fractions (Albenzio et al. 2012); different initial contents of free amino acids; the ratios of amino acids in each milk; and the rate of proteolysis velocity in milk from these ruminants.

The high tyramine level in both products at the end of the 10-day storage period may be attributed to production of free tyrosine, which is further decarboxylated by microbial enzymes to produce tyramine (Özdestan and Üren 2010). Values of 100–800 mg tyramine have been reported as toxic doses in food (Silla-Santos 1996). The values found in this study (560 mg kg^−1^ means for both fermented milks) are within this range of toxicity. Therefore, the ability of these probiotic cultures to produce biogenic amines could be considered a contrastive feature to the beneficial dietary effect on human health.

**Figure 3:**
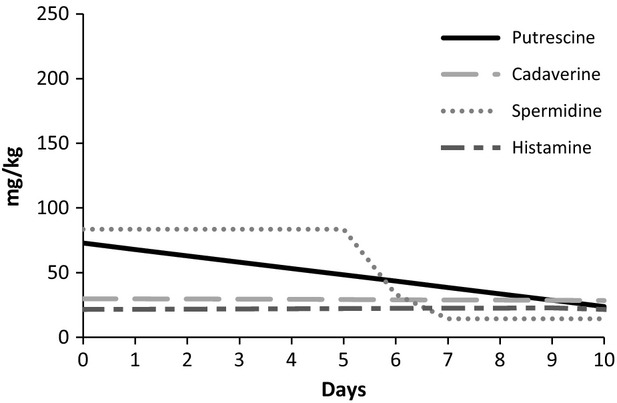
Biogenic amines (putrescine, cadaverine, spermidine, and histamine) behavior found in cow's fermented milk during 10 days of storage.

**Figure 4:**
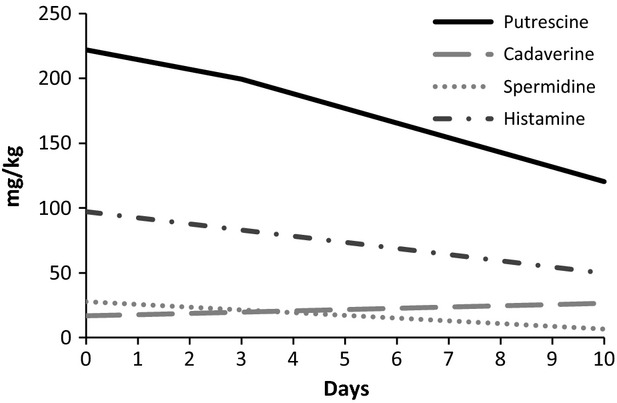
Biogenic amines (putrescine, cadaverine, spermidine, and histamine) behavior found in goat's fermented milk during 10 days of storage.

The fermented cow's milk showed a lower putrescine concentration compared with fermented goat's milk. At day 0, the putrescine content was 83.29 and 104.09 mg kg^−1^, and on day 10 was 20.26 and 22.92 mg kg^−1^, in fermented cow's and goat's milk, respectively (Figs.3, 4). In goat cheese, putrescine increases at the beginning of ripening, followed by a slight decrease (Novella-Rodríguez et al. 2002, 2004). These reductions in biogenic amine contents could be attributable to the ability of some lactic acid bacteria to degrade biogenic amines by means of an enzymatic pathway regulated by oxidase enzymes (Dapkevicius et al. 2000; Tosukhowong et al. 2011).

In respect to cadaverine, the mean concentrations remained constant during storage, 29.09 mg kg^−1^ for fermented cow's milk and 22.07 mg kg^−1^ for fermented goat's milk. These results might be related to the ability of lactic acid bacteria to produce small amounts of cadaverine. Other studies have reported that some strains of lactobacilli (Lorencová et al. 2012) and *S. thermophilus* (Gezginc et al. 2013) can produce cadaverine in dairy products. The presence of putrescine and cadaverine in food could pose an indirect risk to consumers, since they may potentiate the toxicity of other biogenic amines such as tyramine and histamine, by inhibiting the detoxifying enzymes (Flick et al. 2001).

Histamine evolution (Figs.3, 4) was different in fermented cow's and goat's milk. In the former, the histamine concentration (mean 17.97 mg kg^−1^) was constant, while in the latter the histamine level dropped during storage (from 99.06 to 53.85 mg kg^−1^). The initial concentration of histamine in fermented goat's milk was high, approximately 100 mg kg^−1^, which may account for the difference between the histidine concentrations in the two fermented milks (Ceballos et al. 2009). The presence of histamine in food is a public health concern because of its physiological and toxicological effects, which are the most notorious foodborne intoxications. However, the toxicological level of amines is very difficult to establish once it depends on the presence of other amines and individual characteristics (Silla-Santos 1996). According to Parente et al. (2001), histamine intake ranged within 40–100 mg and higher than 100 mg can cause, respectively, intermediate and intensive poisoning.

Spermidine levels behaved similarly in both fermented milks, remaining constant until the fifth day and then decreasing. However, the spermidine concentration was higher in fermented cow's milk (82.07 mg kg^−1^) compared to fermented goat's milk (34.85 mg kg^−1^). Recent studies have demonstrated that strains of *Lactobacillus plantarum* are capable of degrading certain biogenic amines, such as putrescine, spermidine. and histamine (Tosukhowong et al. 2011). Perhaps other strains, as used in this study, may also have the same potential, which would explain the decreases in putrescine, spermidine, and histamine over the storage period.

This study evaluated the production of biogenic amines over a 10-day storage period. This short period is important because the viability of the probiotic culture is highest during this time (Kongo et al. 2006). Compared the results obtained from this study (goat's and cow's fermented milks) with reported by the Özdestan and Üren (2010) in kefir, the values were higher. This difference may be related to the rate of proteolysis velocity in each milk product; presence of microorganisms positive decarboxylase; and expression of the enzyme decarboxylase. However, further research over the entire storage period of probiotic fermented milks is needed, for qualitative and quantitative monitoring of the biogenic amines formed.

### Consumer test

The consumer test was performed in order to verify the sensorial acceptation of the produced fermented milks (probiotic cow's and goat's fermented milks). Fermented cow's milk received a mean score of 5.575, significantly (*P* < 0.05) higher than the score of 2.925 for fermented goat's milk. The best acceptance of fermented cow's milk should be related to the fact that cow's milk is the most produced and consumed from the milk of various species. On the other hand, the characteristic “goaty” taste of goat's milk is unacceptable to many consumers (Slacanac et al. 2010); in a study in the United Kingdom, goat's milk was described as “strong, smelly, salty or sweet” (Mowlem 2005). These intrinsic sensory characteristics are related to the presence of short-chain fatty acids such as caproic, caprylic, and capric acids (Ceballos et al. 2009).

The lower acceptance of fermented goat's milk is in accordance with previous studies of goat cheese and fermented goat milks (Mowlem 2005; Slacanac et al. 2010). The present scores demonstrate the difficulty of producing a goat product with adequate acceptance. One possible alternative to increase the acceptability of fermented goat's milk is the addition of fruit juice and/or pulp. However, addition of fruit juice to probiotic goat's milk yogurt should be carefully evaluated, because inhibitory compounds present in the pulp could decrease the viability of the probiotic strains (Senakaranadheera et al. 2012). Another alternative would be to use new sensory techniques, such as repeated exposure. This methodology can be used to increase the familiarity, and depending on the exposure time, the acceptance of goat's milk products (Costa et al. 2014).

## Conclusions

Even as a first assessment, our findings suggest that processing of probiotic fermented goat's and cow's milks contributes to the formation of biogenic amines during fermentation. Tyramine could be used as a quality index for these fermented milks, because the amount of this biogenic amine was a primary attribute of these fermented milks. Our findings also confirmed that fermented cow's milk is better accepted than fermented goat's milk.
